# A rare case of extra-adrenal pheochromocytoma localized to the ovary and detected via abdominal computed tomography angiography

**DOI:** 10.3892/ol.2014.2718

**Published:** 2014-11-20

**Authors:** HUI LIU, WEN-ZHENG LI, XIAO-YI WANG, YI-GANG PEI, XUE-YIN LONG, CHANG-YONG CHEN, YONG-BIN HU

**Affiliations:** 1 Department of Radiology, Xiangya Hospital, Central South University, Changsha, Hunan 410008, P.R. China; 2 Department of Pathology, Xiangya Hospital, Central South University, Changsha, Hunan 410008, P.R. China

**Keywords:** pheochromocytoma, paraganglioma, extra-adrenal, ovary, computed tomography angiography

## Abstract

Extra-adrenal pheochromocytomas are rare tumors that originate from the chromaffin tissue of the sympathetic nervous system. Ovarian extra-adrenal pheochromocytoma is even more rare. The present study reports a rare case of an extra-adrenal pheochromocytoma that was localized to the right ovary, but was gynecologically asymptomatic. Computed tomography angiography (CTA) detected the tumor and indicated that it was well defined, highly vascularized and obtained its blood supply from the right ovarian artery. This is the second case of ovarian extra-adrenal pheochromocytoma reported in the literature, and the first description of the CTA manifestations in the ovary. Gynecologists and radiologists should consider the possibility that an ovarian mass could be an extra-adrenal pheochromocytoma, which would allow time to prepare appropriately for the surgical removal of the mass.

## Introduction

Pheochromocytomas (also known as paragangliomas) are functional or non-functional tumors of the sympathetic nervous tissue (1). Catecholamine is secreted by functional pheochromocytomas, resulting in paroxysmal hypertension and palpitation. The majority of pheochromocytomas are derived from the chromaffin tissue of the adrenal medulla (2). Extra-adrenal pheochromocytomas are rare tumors that originate from the chromaffin tissue of the sympathetic nervous system (3). In adults, pheochromocytomas are often called the ‘10% tumor’, as ~10% occur above the diaphragm, while 10% of intra-abdominal pheochromocytomas are extra-adrenal, 10% are bilateral, 10% are multiple, 10% are familial, 10% are malignant and 10% recur post-operatively (3). Diagnosing extra-adrenal pheochromocytomas is challenging due to their low incidence, and as their clinical manifestations can be inconsistent. The present study reports an extra-adrenal pheochromocytoma that was localized to the ovary, presented as gynecologically asymptomatic and was detected via computed tomography angiography (CTA). Written informed consent was obtained from the patient.

## Case report

A 43-year-old female with abdominal pain was referred to Xiangya Hospital (Changsha, Hunan, China) and underwent abdominal CTA with a suspected diagnosis of an abdominal aortic dissecting aneurysm. A right ovarian tumor was found, and no dissecting abdominal aortic aneurysm was detected.

The patient’s history included three viable pregnancies, regular menstrual cycles and no history of vaginal bleeding. For the past seven years she had experienced sustained hypertension, with the highest blood pressure recorded being 200/100 mmHg. The patient had not taken any medication to treat the hypertension until one month prior to the present hospital visit for abdominal pain. No headache, syncope or palpitations were indicated.

**Figure 1:**
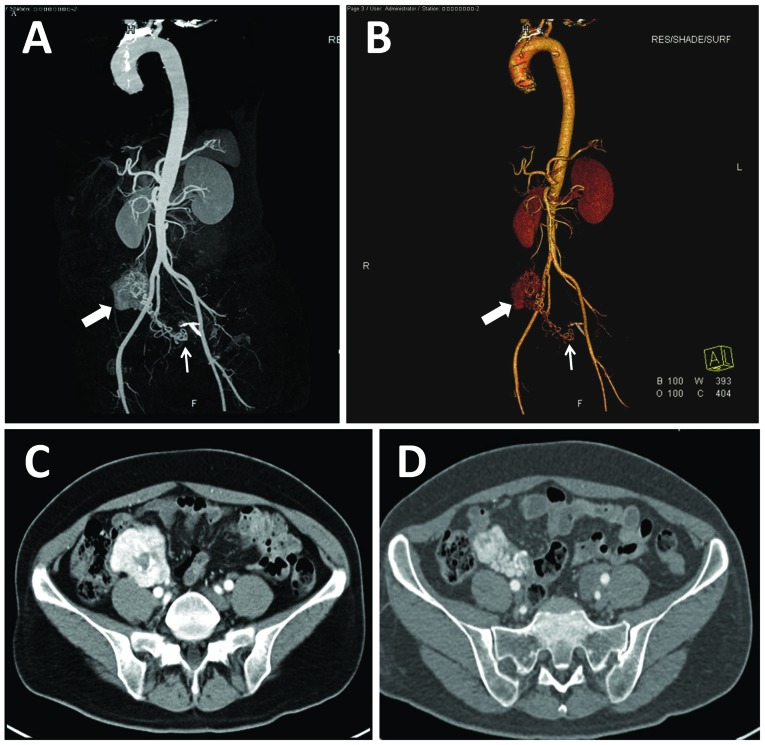
Computed tomography angiography (CTA) of an extra-adrenal pheochromocytoma. Abdominal aortic CTA post-processing: (A) maximum intensity projection and (B) shaded surface display show a highly vascularized tumor (thick arrow). The blood supply of the tumor is derived from the right ovarian and right internal iliac arteries (thin arrow). (C and D) Contrast axial CT demonstrates the tumor location at the superior surface of the right ovary, with clear borders.

Upon physical examination, the patient has a body temperature of 36.4°C (normal range, 36.3–37.2°C), with a heart rate of 106 beats/min (normal range, 60–100 beats/min), a respiratory rate of 20 breaths/min (normal range, 12–20 breaths/min) and a blood pressure of 167/114 mmHg (normal range, 60–90/90–114 mmHg). The gynecological examination indicated a normal uterus and cervix, and no significant mass was palpable. Abdominal CTA showed a well-defined 5.2×5.7×4.0-cm tumor in close proximity to the right ovary (Fig. 1A–D). The tumor was highly vascularized and obtained its blood supply from the right ovarian artery. There was no evidence of hydronephrosis or retroperitoneal lymph node enlargement. Laboratory results indicated a significantly elevated total urinary vanillylmandelic acid (VMA) of 111.8 μmol/24 h (normal VMA, <68.6 μmol/24 h). The patient was thus diagnosed with ovarian extra-adrenal pheochromocytoma.

**Figure 2:**
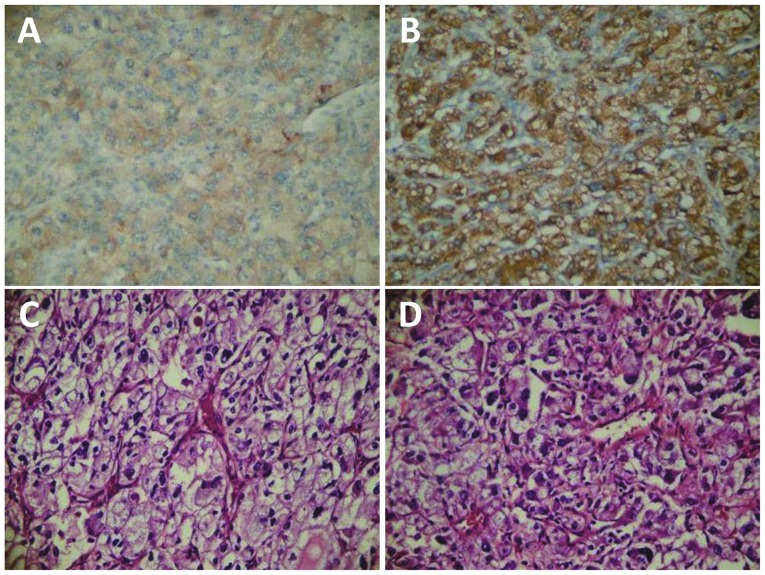
Immunohistochemical and histological staining of the extra-adrenal pheochromocytoma. The tumor was positive for (A) chromogranin A (magnification, ×200), (B) hematoxylin-eosin (magnification, ×200), (C) neuron-specific enolase (magnification, ×200) and (D) synaptophysin (magnification, ×200).

Surgical resection of a 5×5×6-cm mass located superior to the right ovary was performed. Immunohistochemically, the tumor cells were positive for chromogranin A, neuron-specific enolase and synaptophysin (Fig. 2), and the definitive diagnosis was made upon examination of this staining. Following surgery, the patient’s blood pressure stabilized to 115/95 mmHg, with no significant fluctuations, and abdominal pain was no longer experienced.

## Discussion

The majority of extra-adrenal pheochromocytomas are located in the uterus, retroperitoneum, lumbar spine, bladder, ocular orbit, heart and mediastinum (4–8). To the best of our knowledge, the present study is only the second reported case of an ovarian extra-adrenal pheochromocytoma in the literature. Montemurro *et al* (3) reported a case of an ovarian extra-adrenal pheochromocytoma removed via laparoscopic surgery, but there have been no studies describing the CTA manifestations in the ovary. Apart from the extremely rare location of the pheochromocytoma, the present case is significant due to its asymptomatic course and characteristic radiological features.

Extra-adrenal pheochromocytomas are divided into two categories, namely, functional and non-functional tumors. The clinical manifestations of the former are similar to those of adrenomedullary pheochromocytomas. In functional tumors, increased catecholamine secretion is responsible for symptoms such as hypertension, sweating, headache, diaphoresis, anxiety, tachycardia and/or palpitations (9). In addition, the majority of patients with abdominal paragangliomas exhibit abdominal pain or a palpable abdominal mass (10); however, atypical symptoms may also be present. In the current study, the patient presented with abdominal pain similar to that associated with aortic dissection and persistent hypertension. The significantly increased secretion of catecholamine causes the abdominal aorta to contract, leading to abdominal pain. CTA was able to show the tumor site, borders, size and blood supply, which assisted in guiding the surgical plan.

Although rare, the possibility of pheochromocytoma should be included in the differential diagnosis of ovarian tumors, particularly in females with hypertension. Further examinations, such as tests for hematuria VMA, are necessary. Surgical resection is the only treatment option for extra-adrenal pheochromocytomas (11,12). Gynecologists should consider the possibility of an extra-adrenal pheochromocytoma prior to preparing to surgically remove a pelvic mass.

It can be difficult to observe any histological distinction between benign and malignant cases, and therefore predict malignant potential, due to the variable and non-specific clinical presentation and imaging data of these patients. Local recurrence or distant metastases are common. The establishment of an effective diagnosis, management strategy and follow-up regime is vital and requires a multidisciplinary approach involving collaboration between geneticists, endocrinologists, anesthesiologists, surgeons, laboratory specialists, oncologists, radiologists and pathologists.

In conclusion, although ovarian paragangliomas occur infrequently, it is important that they are recognized as a potential cause of an abdominal mass. Clinical and imaging data of patients with extra-adrenal, intra-abdominal paragangliomas are inconsistent. These tumors can even be asymptomatic, as shown in the present case. Therefore, it is essential that a comprehensive histological and immunohistochemical evaluation is performed as the only safe modality for diagnosis. Since these tumors can localize to the ovary and are difficult to definitively diagnose pre-operatively, gynecologists and radiologists should consider the possibility of an extra-adrenal pheochromocytoma prior to preparing to surgically remove a pelvic mass.
